# Competitive fitness analysis using Convolutional Neural Network

**DOI:** 10.21307/jofnem-2020-108

**Published:** 2020-11-06

**Authors:** Joanna K. Palka, Krzysztof Fiok, Weronika Antoł, Zofia M. Prokop

**Affiliations:** 1 Institute of Environmental Sciences, Faculty of Biology, Jagiellonian University, Kraków, Poland; 2 Department of Industrial Engineering and Management Systems, University of Central Florida, Orlando, Florida

**Keywords:** *Caenorhabditis*, Competitive fitness, Convolutional neural network, Fitness method

## Abstract

We developed a procedure for estimating competitive fitness by using *Caenorhabditis elegans* as a model organism and a Convolutional Neural Network (CNN) as a tool. Competitive fitness is usually the most informative fitness measure, and competitive fitness assays often rely on green fluorescent protein (GFP) marker strains. CNNs are a class of deep learning neural networks, which are well suited for image analysis and object classification. Our model analyses involved image classification of nematodes as wild-type vs. GFP-expressing, and counted both categories. The performance was analyzed with (i) precision and recall parameters, and (ii) comparison of the wild-type frequency calculated from the model against that obtained by visual scoring of the same images. The average precision and recall varied from 0.79 to 0.87 and from 0.84 to 0.92, respectively, depending on worm density in the images. Compared with manual counting, the model decreased counting time at least 20-fold while preventing human errors. Given the rapid development in the field of CNN, the model, which is fully available on GitHub, can be further optimized and adapted for other image-based uses.

Fitness, the currency of natural selection, is a fundamental concept in evolutionary biology. Estimating the relative fitness of different individuals and/or populations is a key part of many evolutionary studies. Relative fitness strongly affects the extent to which evolutionary dynamics can be understood and predicted. Relative fitness can be calculated as competitive fitness when two or more genotypes are allowed to compete. However, direct competition between individuals or populations of interest (e.g., evolved vs. ancestral populations in experimental evolution studies) is often impractical because of the difficulty, or even impossibility, in distinguishing their progeny in the population. Thus, relative fitness is often assessed by competition between a population of interest and a common ‘tester’ strain with distinct morphology (reviewed in Teotónio et al., 2017).

*Caenorhabditis* nematodes are increasingly commonly used models in evolutionary and ecological studies, which enable a wide array of questions to be answered (Gray and Cutter, 2014; Teotónio et al., 2017; Cutter et al., 2019). Manual methods of assessing the fitness of individuals or groups of animals (Estes and Lynch, 2003; Estes et al., 2004; Baer et al., 2005; Chelo, 2014; Fritzsche et al., 2014) are extremely time- and energy-consuming. Methods that allow for high-throughput fitness analysis and limit the potential for human bias are therefore strongly desirable. To date, automation of the counting process can be achieved in two ways, both of which are applicable to competitive fitness assays featuring fluorescently marked competitor strains. First, flow-based systems, called ‘worm sorters,’ are used to measure the length, fluorescence emission, and optical density of worms suspended in a fluid (Crombie et al., 2018). The main drawback of such systems is the large initial investment. Another more financially accessible option is image analysis. Image analysis software tools can be used to avoid the need for manual worm counting, thus greatly increasing sample handling speed. Their advantage over worm sorters is the lower investment: to obtain pictures of animals, only a microscope with fluorescence emission and a camera are needed. Image-based analysis also allows for detection of more complex phenotypes than ‘worm sorters’ and for visual verification of the counting software’s performance.

Much progress has been made in this area since the publication of ‘toolbox for high-throughput *C. elegans* assays’ in a software program called CellProfiler (Wählby et al., 2013). Among other uses, this program can be applied to measure the relative fitness of *Caenorhabditis* worms in competition with a GFP (green fluorescent protein)-marked reference strain. In a recent study, Crombie et al. (2018) developed a protocol for assessing the competitive fitness of individual *C. elegans* hermaphrodites by using CellProfiler. In this procedure, one focal and one GFP hermaphrodite are kept together for 7 days to produce offspring. The resulting population is washed from the competition plate and, after several additional steps (see Crombie et al., 2018), is transferred into a 96-well plate, of which two separate pictures (bright-field and GFP fluorescence image) are taken. To count the number of animals, bright-field and GFP fluorescence images are compared by combining the images into one picture and scoring the fluorescing and non-fluorescing animals with CellProfiler (analysis of one image requires approximately 10-30 sec). However, in the time needed for the second picture to be taken, animals change their positions, and misalignment of the two pictures can cause incorrect scoring of animals. To avoid this problem, the movement of animals is hampered with levamisole, a nicotinic acetylcholine receptor antagonist causing paralysis followed by relaxation and death (Crombie et al., 2018). Even with levamisole, animals can still sometimes move. Additionally, the usage of drugs might influence several visible GFP nematodes if some animals die before the pictures are taken, because the expression of GFP is visible in only live animals. Another difficulty in using CellProfiler software is that it scores fragments of animals present at the picture edges. This becomes a problem when animals used in competitive fitness estimations express GFP in only one particular part of the body (e.g., the pharynx). Animals present on the image edges might be assigned to an incorrect category if only the non-glowing part of the animal is visible, thus causing a bias toward non-GFP animals. Finally, the competitive fitness assay protocol described by Crombie et al. (2018) would be problematic to use for the analysis of population-level fitness, in which a larger number of parents together with offspring would bias fitness analysis. To avoid this bias, the offspring generation must be separated from the parents; this can be achieved when the offspring are in the larval stage by sieving them through a filter that lets the larvae, but not the adults, though.

To further improve *Caenorhabditis* fitness analysis, here we present (i) a fitness assay protocol involving separation of parental and offspring generations and (ii) an open-source model for image analysis based on a convolutional neural network (CNN). Machine learning models are very convenient for image analysis because they can be trained on a sample of pictures to analyze images of interest with a high output rate. The concept of analyzing images with CNNs originated in 1968, when Hubel and Wiesel (1968) found that some neurons in the visual cortex in monkeys respond not to the whole visual field but only to its parts. In contrast to a fully connected artificial neural network, CNNs benefit from this observation and connect neurons of one layer to only part of the neurons available in the next layer. If a CNN is provided with an image input, the locally connected layers extract partial information about the analyzed image, which is further used to create an image-level generalization in the final fully connected layers. Over the past several years, the number of image analysis tasks in the field of biology that have been addressed with CNNs has increased. With an open-source CNN architecture, the application of a CNN to a given domain entails the following steps:

1. Preparation of data, in this case, images with marked positive examples; i.e., only objects of interest are highlighted. This process is often referred to as annotation of the data set and is often performed manually by specialists in the field. At this stage, exclusive parts of the data set, e.g., training and validation sets, can be defined.
2. Learning of the CNN model on the training part of the prepared data. The annotated images are fed as input into the CNN. The model attempts to find values for millions of parameters of connections between layers of the neural network that will provide the desired previously annotated output. The trained parameters are often called weights. This learning process is time consuming and is often referred to as training a CNN model. After this step, the model parameters are frozen and are no longer subject to change. The model is then ready to be deployed for classification.
3. Validation of the trained CNN. The trained model is asked to perform classification of images that were not presented to it during training. Predictions of the model regarding images are then compared with the annotations created in step 1. The differences between detected and annotated objects allow for computation of metrics that enable assessment of model quality.

Limited access to annotated, high-quality data are often described as a limitation for new applications of this machine learning technique. In the study of Yang et al. (2016), 393,703 annotated examples of human faces are available, thus demonstrating that vast amounts of data are required for a model to achieve high performance under various weather, light, color, object size and other image related conditions. However, when the domain of microscopic images is addressed, various studies (Gummeson et al., 2017; Wang et al., 2019; Manda-Handzlik et al., 2020) have demonstrated that even small datasets can be sufficient for a CNN model to obtain high-quality results. Our work aligns with the aforementioned research and demonstrates that, in the replicable conditions of microscopic imaging, good performance with dramatically fewer annotated objects can be achieved.

Our model was trained to score worms at early (L1-L2) larval stages, categorizing them as fluorescent (GFP-expressing) vs. non-fluorescent (henceforth called GFP vs. non-GFP). As input, the model used pictures from a fluorescence microscope, with both fluorescent (GFP) and non-fluorescent (non-GFP) animals visible in the same picture (thus avoiding the need to take two separate pictures and hence all problems associated with animal misalignment and levamisole usage). Animals at the picture edges were omitted because they were not fully visible. The analyses (i.e., classification and counting by the model) required approximately 6 sec per picture, although this time would strongly depend on the computing machine.

The performance of the model was evaluated by (i) calculating recall and precision metrics and (ii) comparing the scores obtained by the model with those obtained by analyzing the same images by eye (henceforth called the ‘by eye’ method). Several errors and their types are additionally discussed. The model and training data set are fully available via GitHub and can be freely used as well as further improved upon and trained for other purposes.

## Materials and methods

### Developing and training the CNN model

A deep learning architecture based on CNN called Mask R-CNN (He et al., 2017) was chosen to address the problem of counting two classes of objects, namely GFP and non-GFP (focal) animals. The chosen architecture was previously implemented in Python and is published under an MIT license (Abdulla, 2017). We adopted the version of the model using the ResNet-101 architecture, because it provides higher performance than the other possible choice, ResNet-50 (He et al., 2016).

To train the model, we created a dedicated data set of 1,024 × 819 px images with by-hand annotations of GFP and non-GFP animals. The data set consists of three parts:

1. Training set of images with worms annotated by rectangles, so called ‘bounding-boxes,’ with the use of labelImg (Tzutalin, 2015) software. The annotations are provided in common Pascal VOC (Everingham et al., 2010) and MS COCO (Lin et al., 2014) formats.
2. Training set of images with worms annotated for the instance segmentation task (the boundaries of each worm were precisely marked) in VGG Image Annotator v. 2.0.2 (Dutta and Zisserman, 2019). These annotations are also provided as separate masks in.jpg files.
3. Validation set of images with worms annotated by bounding-boxes for the purpose of assessing model quality.

The first bounding-box part of the training set consisted of 29 original images with 837 non-GFP and 401 GFP annotated animals. The second part of the training set contained 19 images with 501 non-GFP and 334 GFP animals with precisely marked borders. The validation set of images prepared for assessment of model performance after training consisted of 30 images with 850 non-GFP and 507 GFP animals. For all images, we decided not to label animals partially ‘cut’ by image borders, because GFP animals could potentially be misinterpreted as non-GFP because their fluorescent throats might not be present in the picture.

The CNN model was trained on a single GPU Tesla K80 12 GB RAM. We used transfer learning; i.e., our training procedure began not from randomly initialized model parameters but from ResNet-101 weights trained previously on the MS-COCO dataset (Abdulla, 2017). This approach overcame the problem of small training data sets and also greatly decreased training time. Our CNN model had more than 100 neural layers (Abdulla, 2017), distinguished logical sub-entities of the whole model as ‘heads,’ ‘4 +,’ ‘all,’ and consequently trained greater parts of the model before the final step of training all neural layers. Precise definitions of groups of model layers are provided in Abdulla (2017). In our experiments with that approach, the final training procedure required less than 24 hr and was performed in the following manner:

1. first training the model with use of the bounding-box part of our data set for 50 epochs on ‘heads’ model layers, for another 50 epochs on ‘4 +’ layers and for the last 50 epochs on all layers; and
2. further training the model on our training data set with precisely annotated borders of animals for another 25 epochs on ‘heads’ layers, 25 epochs on ‘4 +’ layers and 75 epochs on ‘all’ layers.

During both stages of training, we decided to artificially increase the number of training examples with so-called image augmentation, i.e., artificial modifications applied to original images. The rationale for this approach is that some conditions in which real objects might be visible in images are not represented in the original training data; therefore, artificially creating images with such conditions is beneficial. We decided to use affine transformations, because worms can be seen in various angular orientation, and to use some light-modifying effects to counter the problem of possible differences in light conditions of images taken at various times. For this purpose, we used image augmenters provided in Jung et al. (2020). The description of all model parameters adopted during training and later classification is provided along with the code at https://github.com/krzysztoffiok/c_elegans_fitness.

**Figure 1:**
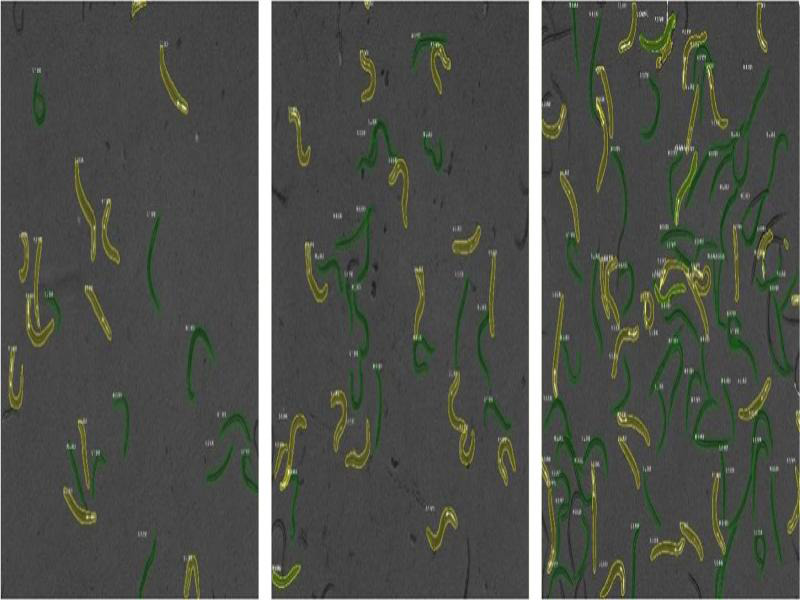
Images with GFP (yellow) and non-GFP (green) animals marked by the CNN model.

When the trained CNN model is used for detection of objects in images, it first determines regions of interest, i.e., looks for regions in the entire image where potential objects appear. Second, in each region of interest, the model attends to classify the object by computing the probability that the given object belongs to the object class it was trained to detect. If, for a given object, the predefined threshold probability value is exceeded, the model returns information regarding the object, i.e., its class and location in the image. The model outputs a .csv file containing the name of the image with a size of classes (GFP and non-GFP animals) and pictures with precisely segmented animals, and an additional .csv file with their locations described by bounding boxes. Examples of obtained images with animals assigned to categories are shown in Figure 1.

**Figure S1:**
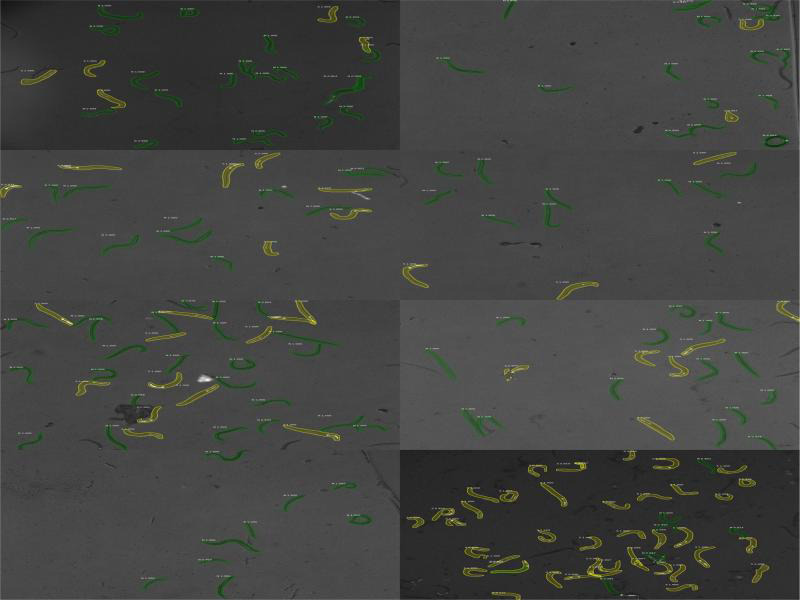
Pictures that rendered: the most extreme proportion differences (+) on the first panel, the most extreme proportion differences (−) second panel, no difference on the last panel.

After training, the CNN model was tested on the validation part of the data set to compute values of metrics from the image-analysis domain, i.e., the average precision and average recall, as proposed in The PASCAL Visual Object Classes Challenge (Everingham et al., 2010). Precision was defined as the sum of true positive predictions divided by the sum of all true positive and false positive predictions made by the model. The recall was considered the sum of true positive model predictions divided by the sum of animals that a human observer (JP) labeled as true positive. Because the aim of this image analysis was counting actual worms, a moderate level of correctness of localization of the worms found by the model was adopted; i.e., the threshold Intersection over Union (IoU) value was set to 0.5. For each worm, the IoU was defined as the intersection of the area marked by the human annotator and by the model, divided by the area of the union of these areas. To test whether the density of animals affected the function of the model, we calculated the metrics separately for densities below and above 70 animals.

To gain further insight into the model’s performance, we used the concept of computing metrics for three groups of object sizes proposed in the MS-COCO competition (Lin et al., 2014). The objects were divided into three size groups: smaller than 32 square pixels, between 32 and 96 square pixels, and larger than 96 square pixels.

### Competitive fitness assays and their analysis with the model

*C. elegans* strain N2 with the *fog-2 q71* mutation was used as a focal strain (henceforth called focal). A marker strain with bright GFP expression in the pharynx (created in our lab by introgressing the GFP allele from *C. elegans* strain PD4792 onto the genetic background of N2 strain) was used as a ‘tester’; henceforth called GFP. We set up competition experiments in which pre-defined (estimated, described below) numbers of focal and GFP worms were seeded on plates and allowed to reproduce. Subsequently, samples of their offspring, separated from the parental generation, were mounted on slides, and multiple non-overlapping pictures under fluorescence were taken from each slide and fed into the model, which scored focal and GFP worms visible in each picture. To assess the model performance over a wide range of ratios of focal: GFP individuals present in images, we used fitness assays with three treatments, each with a different proportion of focal individuals in the parental generations: 0.45, 0.60, and 0.75. This process resulted in estimated focal proportions in the offspring generation ranging from 0.13 to 0.79 at the mixed population level, and from 0 to 1 at the level of individual images.

### Competitive fitness assay protocol

The 14 cm ø (diameter) agar plates with strains of interest (focal/GFP) were washed with 4 ml of S-Basal solution (Stiernagle, 2006). Animals suspended in liquid were placed on a nylon filter with 15 µm diameter mesh placed on top of a plastic vial. The filter let through L1 and L2 larvae and was used to separate adult worms from their offspring. Larvae present in 2 to 3 drops of 1 µl were counted. According to this count, the volume of liquid required to obtain a desirable population size was calculated. Three types of mixed populations were created, with estimated initial numbers of focal vs. GFP individuals of 900 vs. 1,100 (initial focal proportion 0.45; 15 replicate mixed populations set up in this treatment), 1,200 vs. 800 (initial focal proportion 0.6; 15 mixed populations), or 1,500 vs. 500 (focal proportion 0.75; 25 mixed populations), respectively. Animals were placed on 6 cm ø Petri dishes and left for 3 days to mature and produce offspring.

After 3 days, the offspring at the L1/L2 stage were collected from the dish by washing worms off the agar with 1 ml S-Basal buffer and placing the resulting suspension on the 15 µm filter to sieve off the adults (parental generation). The filtered liquid was left for ~15 min to allow the larvae to settle at the bottom and was then transferred onto a glass slide (our laboratory setup required an additional transfer of 300 µl of the suspension into a smaller microcentrifuge tube; however, in other settings, this step may not be necessary). After transferring, a 5 µl droplet onto a glass slide, to reduce the animals’ mobility and maintain even focus, we gently placed a cover slide on the drop surface. From one glass slide, 10 non-overlapping pictures were taken under 40× magnification with a Nikon Eclipse 80i microscope with a BV-1A filter combination (435/10 nm excitation filter, 470 nm barrier filter and dichromatic mirror value 455 nm). Other settings of the microscope included shutter (opened) and contrast (maximum). The microscope was connected to a computer with a Nikon Digital Sight DS-U3 camera. The pictures were taken with NIS-Elements software with the following settings: filter turret: off (black and white pictures), exposure time: 30 ms, grain: 27.

### Analyses of model performance

A total of 550 pictures (150, 150, and 250 from 0.45, 0.6, and 0.75 initial focal proportion treatments, respectively), representing 55 mixed populations, were scored by both the model and a human observer. Visual counting by human observers was done in ImageJ, by marking animals with a multipoint tool. For each picture, the proportion of focal individuals relative to the total number of individuals present in the picture was calculated separately from data collected by the model and by eye. For each mixed population, the proportion of focal individuals was also calculated separately from data collected by the model and by eye; in each case, this process was performed by summing the numbers of focal and GFP individuals from all 10 pictures taken for the population and calculating the fraction of focals among all worms present. Proportion of focal individuals (out of the total sum of focal + GFP individuals) was used as a measure of fitness of the focal population, which is an approach commonly used in competitive fitness experiments (see e.g. Teotónio et al., 2012, 2017; Crombie et al., 2018).

For each mixed population as well as each individual picture, we calculated the difference between the proportions of focal offspring on the basis of scoring by the model vs. by eye (henceforth called proportion difference). To evaluate what properties of the pictures led to discrepancies between model vs. visual scores, we inspected 16 pictures that rendered the most extreme proportion differences (between −0.238 and −0.150, or between 0.200 and 0.289, indicating the proportion of focals being lower or higher, respectively, when calculated from model-scored in comparison to human-scored data), as well as eight pictures with no difference (cf. Supplement 1). The inspection revealed that (i) large discrepancies between model vs. human scores were in fact due to model rather than human errors, and (ii) model errors were largely due to poor image illumination and/or strong clustering of animals.

To determine which types of errors the model is most vulnerable to and how the number of errors changes with the number of animals present in a picture (in which we expected a positive relationship, because clustering of animals should be more likely with their increasing density), we randomly selected 60 pictures from one of the proportion treatments (proportion of focals in the parental generation=0.75) and performed a careful analysis. Different categories of outcomes were distinguished depending on whether the animal classifications were (i) classified correctly (i.e., GFP as GFP, focal as focal), (ii) classified incorrectly (GFP as focal or vice versa), (iii) counted twice, (iv) missed, or (v) falsely classified as animals. Scores from each outcome were regressed against the number of animals present in a picture with a linear regression model (lm function), performed in RStudio (R version 3.6.2; R Core Team, 2018); *y* = *a* + *bx*, where *y* is the count of the outcomes in question, *x* is the total number of animals present on picture (scored ‘by eye’), whereas *a* and *b* are regression coefficients estimated in the analysis.

Crucially, we asked to what extent the model errors might affect the results of fitness assays. Thus, we regressed the proportion difference against the proportion of focals calculated from data scored ‘by eye’, considering the latter to be our most reliable estimate of the true proportions of focals. Regressions were performed as described above but with proportion difference as the dependent (*y*) and proportion calculated based on scoring images ‘by eye’ as the predictor (*x*) variables. We ran these regressions, taking as data points the proportions and proportion differences scored from either (i) individual pictures or (ii) mixed populations (proportions estimated for each population according to the sample of pictures taken for these populations; see ‘Competitive fitness assay protocol’ section above).

Finally, variability of the two methods (model vs. manual) – the eye and model count was checked. To do this, the proportion of focal animals (*p*) in each mixed population was estimated as the sum of focal animals scored from 10 pictures taken for this population over the total sum of all (focal + GFP) animals scored from the same 10 pictures, and the variability of this estimate was calculated as the standard deviation of the 10 proportions scored for each of the 10 pictures. Subsequently, we compared both the proportions and the SDs between methods using the Wilcoxon paired difference test (since each population was scored using both methods). Additionally, we also checked for the relationship between the proportion estimate and its variability by regressing SDs against proportions, separately for each method.

## Results

### Evaluation of worm detection

Tables 1 and 2 show the values of model performance metrics on the validation set. Both precision and recall parameters had higher values for smaller numbers of animals present in a picture.

**Table 1.** Performance metrics of the CNN model computed on the evaluation set for low and moderate animal densities (below 70).

	Area			
Metric	Small	Medium	Large	All
Average precision @ IoU = 0.50	No animals	0.870	0.883	0.872
Average recall @ IoU = 0.50	No animals	0.930	0.918	0.917

**Table 2.** Performance metrics of the CNN model computed on the evaluation set for high animal densities (above 70).

	Area			
Metric	Small	Medium	Large	All
Average precision @ IoU = 0.50	No animals	0.784	0.810	0.787
Average recall @ IoU = 0.50	No animals	0.851	0.856	0.842

The performance of the model was highly dependent on the lighting conditions. Therefore, in the protocol, we include the exact values of program parameters and microscope settings under which the model was ‘taught’ to recognize individuals. We also include examples of pictures with proper and improper lightning and visibility of GFP nematodes (Supplement 1).

### Types of error

**Figure 2:**
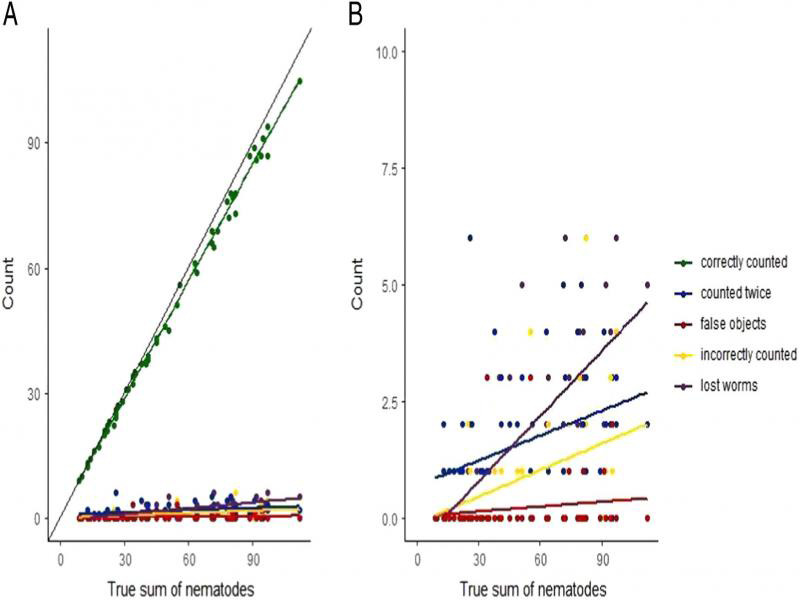
(A) Correct detection of worms and the number of errors at increasing concentrations of animals, (B) Close-up of error types at increasing animal density.

As shown in Figure 2B, together with the increasing density of animals, the number of errors also slightly increased. This small increase was most likely due to the aggregation of animals at higher densities. Analyzed errors were divided into categories: counted twice, false, incorrect count, and lost animals (Fig. 2B; regression slopes: 0.0179, 0.0034, 0.0190, and 0.0469, with *P* values: 0.0063, 0.2378, 0.0005, and << 0.0001, respectively). The regression slope for the number of correctly identified animals was very close to, albeit significantly lower than 1 (Fig. 2A, *b* = 0.9341, SE = 0.0075), indicating that despite some errors, the model correctly recognized most animals across the range of densities, albeit the accuracy did deteriorate with increasing densities.

### Accuracy of fitness estimates

At the level of individual pictures, regression analysis showed a significantly negative relationship (*b* = −0.057, df = 548, *P* = 0.0002) between the proportion of focals calculated on the basis of scoring pictures by eye and the proportion difference, which was calculated by subtracting the proportion of focals calculated from ‘by eye’ scores from the analogous proportion calculated from model scores, and ranged from −0.238 to 0.289. The negative regression slope indicates that for smaller true proportions of focals (assessed by carefully scoring the pictures by eye), the model tends to produce an upward bias, whereas for higher true proportions it tends to produce a downward bias. When analyzed at the population level, the trend remained negative but became flatter and non-significant (*b* = −0.031, df = 53, *P* = 0.429).

### Variability of two methods

**Figure 3:**
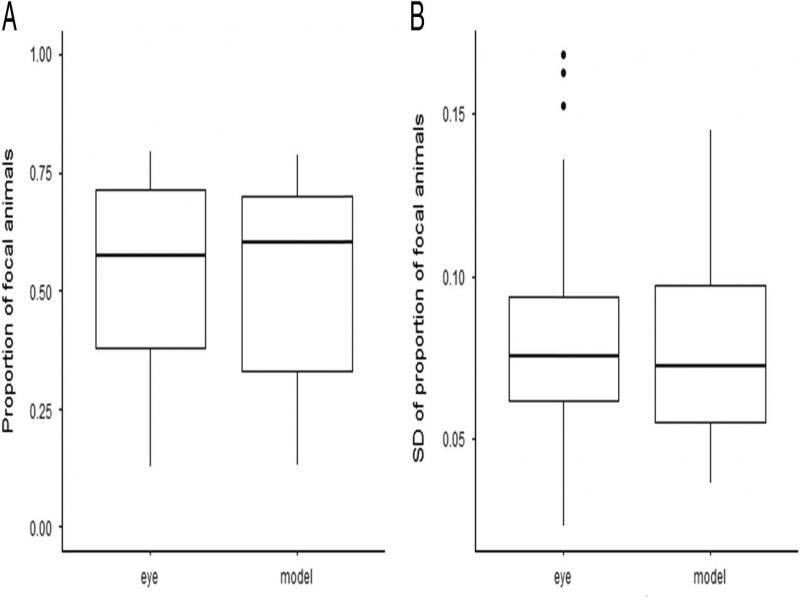
(A) Boxplot of the frequency of focal animals for the two methods, (B) Boxplot of the standard deviation of the proportion of focals for the two methods.

Neither the average proportion of focals, nor its variability at the level of mixed populations (estimated by the SD of proportions scored from all images taken for each population) differed significantly between the two methods (Wilcoxon paired differences test; proportions: *P* = 0.7243, proportion SDs: *P* = 0.6582, cf. Fig. 3). Regression analyses performed separately for the two methods showed moderate relationship between proportion mean and SD (R2 of 0.141 and 0.233 for the ‘by eye’ and model scoring, respectively) (Supplement 2).

**Figure S2:**
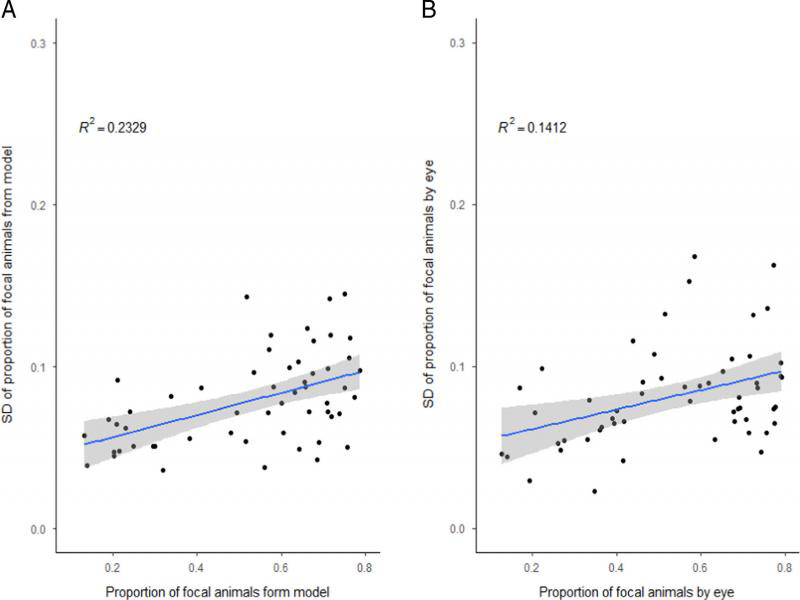
Variability of measures of competitive fitness. Plots of measures of variability (*y*-axis) vs. measures of competitive fitness (*x*-axis). Panel A show the plots for the model, while panel B, for the plots for count ‘by eye’. Panels show the frequency of the focal animals as the measure of competitive fitness.

## Discussion

In evolutionary studies, competitive fitness assays are generally preferable to lifetime reproduction assays (Crombie et al., 2018). In competitive fitness assays of *Caenorhabditis* nematodes, animals of interest are placed with a competitor on an agar plate, and the proportion of offspring produced is calculated. To distinguish competitors from focal offspring, GFP marked nematodes are often used as the former (e.g., Teotónio et al., 2012; Palopoli et al., 2015, reviewed by Teotónio et al., 2017).

Here, we present a machine learning-based approach (along with the experimental protocol) for high-throughput analysis of competitive fitness in *C. elegans*. Beyond strictly practical applications, the additional aim of this study was to demonstrate the possibility and advantages of applying machine learning image analysis techniques in the analysis of the fitness of *C. elegans.*

The comparisons presented in the Results section show that for smaller true proportions of focal animals present in the pictures (which can have values from 0 to 1), the model tends to bias this proportion upward, whereas for higher true proportions, it tends to produce a downward bias. This trend was highly significant (*P* = 0.0002), partly because of a large sample size (550 pictures), yet not very steep (*b* = −0.057). Importantly, the trend became even less steep (*b* = −0.031) when entire population samples (10 pictures taken per population; see Methods) rather than individual pictures were used, emphasizing the importance of taking multiple images per experimental mixed population for obtaining reliable estimates. Furthermore, in actual fitness assays wherein the fitness of different treatment groups or strains is compared, such bias would actually increase conservatism, by making the detection of fitness differences between groups (slightly) more difficult.

### Advantages of the method

Compared with manual counting from images, using the model decreased the counting time at least 20-fold (personal observations). The contrast would be even larger for manual counting with an aspirator, in which researchers may, in our experience, spend vast amounts of time searching for animals hidden in the agar, although in this comparison, the time spent preparing samples and taking pictures should also be considered. In our experience, this procedure requires approximately 50 min per 100 pictures, including all steps (along with washing animals through filters, which would be necessary regardless of the method of counting, as long as the experiment requires separating offspring from parental generation; see below).

Compared with previous automatization protocols using CellProfiler software (Crombie et al., 2018), our method offers several advantages. First, it does not require taking both bright-field and GFP fluorescence images from the same frame; instead, one picture is taken in which GFP animals are distinguished from non-GFP animals by the CNN algorithm. This procedure resolves the problem of misalignment of animals while also not requiring the usage of levamisole, which can affect animals’ mortality and hence GFP expression. Second, our experimental protocol separates offspring from the parental generation; this process is advantageous for population-level fitness assays, wherein large numbers of parents counted along with offspring could substantially bias fitness estimates. Third, the model can be trained for other purposes specific to other studies’ aims (it is fully available via GitHub [https://github.com/krzysztoffiok/c_elegans_fitness]).

### Limitations of the method

For the purpose of *C. elegans* fitness analysis, we consider the model’s performance to be highly satisfactory; nevertheless, it did produce some errors. The main source of error was tangling of animals, which inhibits proper distinction among individuals. Tangling of worms has also been a major problem in previous approaches to image analysis for counting *C. elegans* (Wählby et al., 2013). This problem can be reduced if the densities of animals in the pictures are kept at moderate level. To maintain optimal density of animals in the pictures, during slide preparation, we recommend using an additional dilution (by adding S-Basal onto the surface) for slides with very high worm densities. Other problems include omitting some animals, and incorrect category assignment or double counting of one animal. In addition, sometimes GFP animals with weak fluorescence could be misinterpreted as non-GFP. Therefore, during the procedure, keeping animals alive is extremely important, because dead individuals do not emit fluorescence. It is also important to emphasize that the model presented here was trained with images of *C. elegans* captured in precisely defined conditions; therefore, applying this model to analysis of images captured under different conditions, e.g., lighting conditions, would cause the model to work in an unpredictable manner. Consequently, proper illumination and visible contrast between GFP and non-GFP individuals is crucial. If the lightning conditions are as described in this paper, there should be no need for further model training before analysis. However, any changes in illumination settings or magnification would require further development and implementation of the new training set.

In summary, given the rapid development of the field of CNN, the model’s performance could be further optimized to achieve even better performance. With further development, application of deep learning algorithms could allow this model to be extended to other fields of the biology of *C. elegans* (or other animals). CNN analysis has found a broad range of applications, and it is a powerful tool in image analysis. Despite the described errors, the obtained method provides a good estimate of competitive fitness. It can be effectively used to increase the efficacy of fitness estimations, as compared with that of manual counting.
